# Vectorcardiography for Optimization of Stimulation Intervals in Cardiac Resynchronization Therapy

**DOI:** 10.1007/s12265-015-9615-7

**Published:** 2015-03-06

**Authors:** Caroline J. M. van Deursen, Liliane Wecke, Wouter M. van Everdingen, Marcus Ståhlberg, Michel H. G. Janssen, Frieder Braunschweig, Lennart Bergfeldt, Harry J. G. M. Crijns, Kevin Vernooy, Frits W. Prinzen

**Affiliations:** 1 Departments of Physiology, Cardiovascular Research Institute Maastricht, Maastricht, The Netherlands; 2 Departments Cardiology, Cardiovascular Research Institute Maastricht, Maastricht, The Netherlands; 3 Karolinska Institutet, Department of Cardiology, Karolinska University Hospital, Stockholm, Sweden; 4 Sahlgrenska Academy, Department of Molecular and Clinical Medicine/Cardiology, University of Gothenburg, Gothenburg, Sweden; 5 P.O. Box 616, 6200 MD Maastricht, The Netherlands

**Keywords:** Cardiac resynchronization therapy, Biventricular pacing, Vectorcardiography, Electrocardiography, Atrioventricular timing, Interventricular timing

## Abstract

Current optimization of atrioventricular (AV) and interventricular (VV) intervals in cardiac resynchronization therapy (CRT) is time consuming and subject to noise. We aimed to prove the principle that the best hemodynamic effect of CRT is achieved by cancelation of opposing electrical forces, detectable from the QRS morphology in the 3D vectorcardiogram (VCG). Different degrees of left (LV) and right ventricular (RV) pre-excitation were induced, using variation in AV intervals during LV pacing in 20 patients with left bundle branch block (LBBB) and variation in VV intervals during biventricular pacing in 18 patients with complete AV block or atrial fibrillation. The smallest QRS vector area identified stimulation intervals with minimal systolic stretch (median difference [IQR] 20 ms [−20, 20 ms] and maximal hemodynamic response (10 ms [−20, 40 ms]). Reliability of VCG measurements was superior to hemodynamic measurements. This study proves the principle that VCG analysis may allow easy and reliable optimization of stimulation intervals in CRT patients.

## Introduction

In cardiac resynchronization therapy (CRT), the time intervals between electrical stimulation of the right atrium and ventricles (atrioventricular (AV) interval) and between the right and left ventricle (interventricular (VV) interval) determine left ventricular (LV) filling characteristics and the degree of ventricular resynchronization. Multiple techniques are used for optimization of the AV and VV intervals, such as echocardiography (LV outflow tract velocity time integral, mitral inflow patterns), invasive hemodynamics (LV *dP*/*dt*_max_), and finger blood pressure measurements and electrogram (EGM)-based methods. Acute hemodynamic improvements by up to ~15 % for AV and VV optimization compared to nominal settings have been described [1–4]. However, favorable long-term effects in large clinical trials have not been observed [5–8]. One of the reasons for the absent evidence of long-term benefit might be that the parameters used for optimization have a high measurement variability, leading to a low “signal to noise ratio” [9, 10]. This seems even the case for invasively measured LV *dP*/*dt*_max_, often regarded as the gold standard of hemodynamic response [2]. The lack of evidence for long-term benefit of optimization and the time-consuming nature of the methods used to optimize CRT make it understandable that a vast majority of implanting physicians leave CRT device settings at their nominal values (“out-of-the-box”) [11].

Several algorithms have been introduced to perform CRT optimization in an automated device-based manner. Some algorithms are based on baseline intracardiac EGMs, predicting maximal resynchronization [5, 12]; others use special sensors (accelerometer for determination of peak endocardial acceleration) [13], but clinical benefits have only been demonstrated for the adaptive CRT algorithm in patients where synchronized LV pacing was performed [14].

Vectorcardiography (VCG) contains 3D information of electrical forces within the heart and thus may provide a valuable description of the degree of resynchronization during LV or biventricular (BiV) pacing. We recently showed in ischemic and nonischemic failing canine hearts with left bundle branch block (LBBB) that VCG reflects electrical interventricular dyssynchrony and is a reliable and reproducible tool for AV and VV optimizations [15].

The present study was designed to prove the principle of using 3D VCG for optimization of AV and VV intervals in CRT recipients. We hypothesized that optimal AV and VV intervals are predicted by the smallest QRS vector area (QRSV_AREA_), because it expresses the highest degree of opposition of electrical forces from two directions (“cancelation”) [16], implying the best electrical resynchronization. VCG predictions of optimal AV and VV intervals were assessed in relation to those determined by noninvasive hemodynamic measurements and strain patterns.

## Methods

### Patient selection

The study population consisted of 38 patients, implanted with a CRT device according to current guidelines, with either sinus rhythm and LBBB (*n* = 20; AV group) or complete AV block/atrial fibrillation with a slow ventricular rate (*n* = 18; VV group). Measurements were performed at least 3 months after CRT implantation. In the AV group, we investigated the full range of electrical dyssynchrony by studying activation sequences caused by intrinsic (LBBB) conduction and by LV pacing in combination with various degrees of fusion with intrinsic conduction. Previous studies have shown that by using LV pacing at various AV intervals, all degrees of dyssynchrony can be studied that are achieved by BiV pacing at various AV and VV intervals [17–19]. The VV group was used to investigate the effect of right ventricular (RV) and LV pacing without fusion with intrinsic conduction.

The study was performed according to the principles of the Declaration of Helsinki and approved by the ethics committee of Maastricht University Hospital and the regional ethical review board in Stockholm. All participants gave fully informed written consent prior to investigation.

### Pacing Protocol

In the AV group, single chamber LV pacing was performed during overdrive atrial pacing (80 bpm) at AV intervals ranging from 50 ms until intrinsic conduction (ventricular noncapture) with steps of 20–25 ms. AAI pacing at 80 bpm was used as baseline. In the VV group, BiV pacing was performed at a rate of 80 bpm with VV intervals ranging from 80 ms LV pre-excitation (negative values) to 80 ms RV pre-excitation (positive values) in 20 ms steps, using simultaneous BiV pacing (VV interval of ~0 ms) as baseline. In the patients with complete AV block (*n* = 12), the AV interval was kept constant with the time from paced right atrium to the first paced ventricle (either right or left sided) being 120 ms. The pacing protocols were alternatingly executed in a top-down (starting with short AV delays and maximal LV pre-excitation) or a bottom-up order to minimize potential bias.

### Vectorcardiographic and Electrocardiographic Measurements

VCG was performed using eight electrodes positioned according to the modified Frank orthogonal lead system (*X*, *Y*, and *Z*; Coronet II System, Ortivus AB, Danderyd, Sweden) with patients at rest and in supine position. With each pacing configuration, recordings were made at a sampling frequency of 500 Hz for 3 min and signals were averaged over half a minute. The VCGs were analyzed offline using customized software [20]. The maximum distance between the origin (0,0,0) and a point on the 3D QRS vector loop was represented by the maximal QRS vector amplitude (QRSV_AMPL_ (mV)). The direction of the maximal QRS vector in space was determined using the angle in the transversal plane and the angle in craniocaudal direction. The QRS vector area (QRSV_AREA_ (μVs)) was assessed as the “3D area” between the curve and the baseline from the beginning to the end of the QRS complex in *X*, *Y*, and *Z*, and calculated as (QRS*x* ^2^ + QRS*y* ^2^ + QRS*z* ^2^)^½^.

Because VCG measurements are not standard, we also investigated the possibility to use the standard 12-lead ECG for optimization of CRT. To this purpose, the ECG was recorded for each pacing configuration. Analysis was performed on the ECG lead that showed the largest change in QRS amplitude between LBBB or RV pacing and LV pacing, thereby reflecting the largest projection of the 3D QRS vector loop. For each patient, this lead was defined as lead_MAX_. Based on previous studies in canine hearts [15], we predicted the optimal AV and VV intervals from the QRS amplitude in lead_MAX_ (QRSE_AMPL_), tracing a value closest to that halfway in between fully captured LV pacing and LBBB or RV pacing (∆QRSE_AMPL_HW_). QRSE_AMPL_ was measured manually as the net deflection of the QRS complex, defining rS or QS morphologies as negative and R or Rs morphologies as positive.

### Finger Blood Pressure Measurements

Finger blood pressure measurements were acquired using a plethysmographic approach (Nexfin, BMEYE B.V., Amsterdam, The Netherlands). Systolic blood pressure (SBP) and stroke volume (SV) were derived from the continuous signal. This algorithm has previously been validated for optimization of CRT settings [21]. Each pacing configuration was directly compared to the baseline situation during two transitions (from baseline to a stimulation interval and back). The ten beats before and after each transition were selected and averaged using customized Matlab software (MathWorks, Natick, MA, USA) [21].

### Echocardiographic Measurements

After the simultaneous VCG and finger blood pressure measurements for all pacing configurations, transthoracic echocardiographic recordings were made using an iE33 system with S5-1 transducer (Philips Medical Systems, Best, The Netherlands). LV outflow tract velocity time integral (VTI_LVOT_) was calculated offline for six consecutive beats. Pulsed wave Doppler echocardiography was used to measure interventricular mechanical delay (IVMD) as the difference between time to onset of pulmonary and aortic ejection (a negative IVMD indicating earlier aortic ejection). Apical four-chamber views were obtained with high frame rates (60–90 Hz) during breath-hold at end-expiration for three consecutive beats. In these images, septal strain was determined offline in the middle 50 % of the septal segment using speckle tracking software (QLAB version 8.1, Philips Medical Systems, Bothell, WA, USA). From the septal strain signals, two indices were calculated using customized Matlab software: septal systolic pre-stretch (SPS), defined as early systolic septal stretch and representing late septal activation (septal stretching due to early unopposed contralateral LV free wall contraction), and septal systolic rebound stretch (SRS), defined as systolic stretch following initial shortening and representing early septal activation (septal stretching due to late vigorous contralateral LV free wall contraction) [22]. Such stretch during the systolic phase diminishes effective pumping, since the stretch “absorbs” the shortening of other ventricular wall segments. Consequently, the lowest amount of stretch during systole (SPS + SRS) was considered to represent the most physiological state.

### Determination of the Optimal Hemodynamic AV or VV Interval

The hemodynamic effect of changing the AV or VV interval was defined as the relative change from baseline for the combination of VTI_LVOT_ and finger blood pressure measurements; ∆HEMO (%) = 0.5 × (ΔVTI_LVOT_ + 0.5 × (ΔSBP + ΔSV)). Combining the data from the independently performed finger blood pressure and VTI_LVOT_ measurements can be expected to improve the accuracy of the measurement of the hemodynamic effect [9]. The AV or VV interval with the largest ∆HEMO was considered the optimal interval. All assessors were blinded to the results from the other methods.

### Statistical Analysis

Statistical analyses were performed using IBM SPSS Statistics software version 20 (SPSS Inc., Chicago, IL). Continuous variables were presented as mean ± SD or as median and interquartile range (IQR; 25th–75th percentile) in case of nonnormal distribution, categorical variables as number (percentage). Mutual linear correlations between different parameters at the various AV and VV intervals were evaluated by Pearson’s correlation coefficient. Agreement between the various predictions of optimal AV and VV intervals was evaluated with a Bland-Altman analysis, containing the mean difference (bias) and the limits of agreement (defined as mean ± 1.96*SD) [23]. The goodness of fit of raw data points at the various AV and VV intervals with a fitted sixth order polynomial curve (expressed as *R* ^2^) was used as an indicator of reliability of the measurements (less noise in a measurement will produce a better fit).

## Results

Clinical characteristics are presented in Table 1 and are fairly representative for a CRT population.

**Table 1:** Baseline characteristics

Patient characteristics, *n* (%) (mean ± SD)	AV group (*n* = 20)	VV group (*n* = 18)	Total (*n* = 38)
Age (years)	[64.2 ± 8.2]	[68.8 ± 8.9]	[66.3 ± 8.7]
Male gender	20 (100 %)	14 (78 %)	34 (89 %)
Ischemic HF etiology	8 (40 %)	10 (56 %)	18 (47 %)
Pre-CRT values			
QRS duration (ms)	[167 ± 20]	[177 ± 19]	[172 ± 20]
LBBB morphology	19 (95 %)	4 (22 %)	23 (61 %)
RV pacing/AVB	0 (0 %)	13 (72 %)	13 (34 %)
LVEF (%)	[25 ± 7]	[26 ± 10]	[25 ± 9]
LVESV (ml)	[187 ± 71]	[154 ± 62]	[171 ± 68]
CRT responders	15 (75 %)	10 (56 %)	25 (66 %)
RV lead position			
RV outflow tract	5 (25 %)	1 (6 %)	6 (16 %)
Septum	0 (0 %)	1 (6 %)	1 (3 %)
Apex	15 (75 %)	16 (89 %)	31 (82 %)
LV lead position			
Anterior	0 (0 %)	1 (6 %)	1 (3 %)
Anterolateral	8 (40 %)	5 (28 %)	13 (34 %)
Lateral	3 (15 %)	1 (6 %)	4 (11 %)
Inferolateral	8 (40 %)	8 (44 %)	16 (42 %)
Inferior	1 (5 %)	3 (17 %)	4 (11 %)
Medication			
β-Blocker	20 (100 %)	17 (94 %)	37 (97 %)
ACE-inhibitor/ARB	19 (95 %)	18 (100 %)	37 (97 %)
Loop diuretics	10 (50 %)	12 (67 %)	22 (58 %)
Aldosterone antagonist	9 (45 %)	10 (56 %)	19 (50 %)
Amiodarone	1 (5 %)	1 (6 %)	2 (5 %)

CRT response was defined as a ≥5 % point improvement in LVEF after 3–6 months of CRT determined by echocardiography according to the biplane method of disks (modified Simpson)

*HF* heart failure, *LBBB* left bundle branch block, *RV* right ventricle, *AVB* complete AV block, *LVEF* left ventricular ejection fraction, *LVESV* left ventricular end systolic volume, *NYHA* New York Heart Association functional class, *CRT* cardiac resynchronization therapy, *LV* left ventricle, *ACE* angiotensin-converting enzyme, *ARB* angiotensin II type 1 receptor blocker

### Magnitude and Direction of the QRS Vector During LBBB, RV Pacing, and LV Pacing

**Fig. 1:**
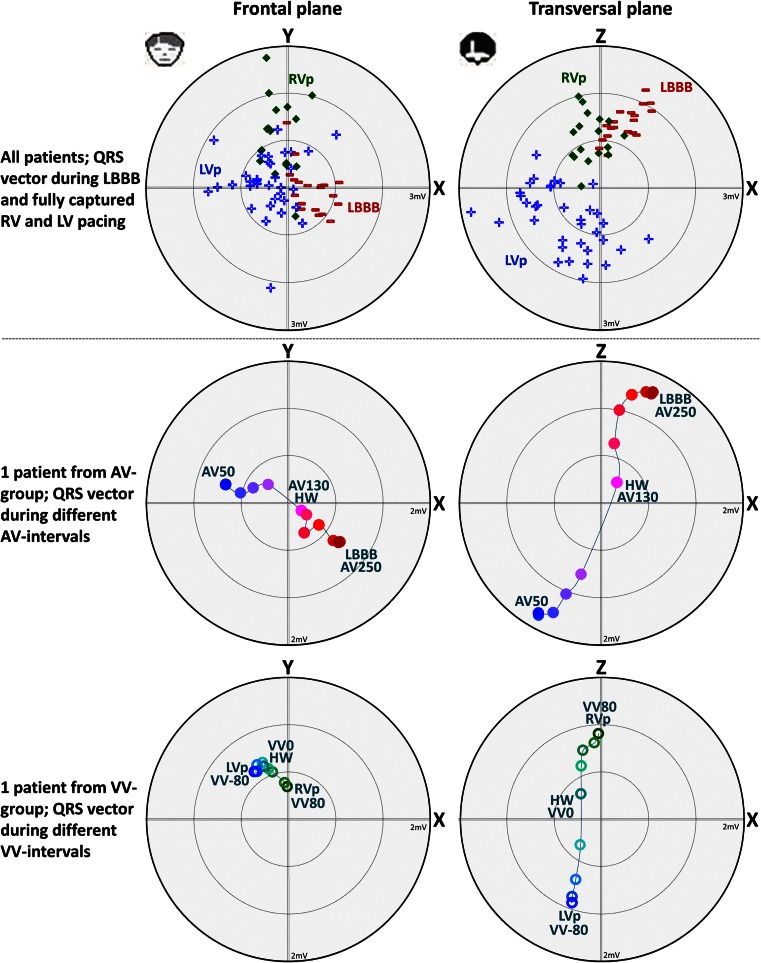
QRS vector amplitude and direction for the different pacing configurations. *Upper row*: QRS vectors of all patients for intrinsic conduction (LBBB; *hyphen*; *n* = 20), RV pacing (*diamond*; *n* = 18), and LV pacing (*plus sign*; *n* = 38) in the frontal (left) and transversal (right) plane. *Middle row*: QRS vector amplitude (QRSV_AMPL_) for a representative patient of the AV group during different AV intervals with indication of the halfway (HW) value of QRSV_AMPL_ in between LV pacing and LBBB. *Lower row*: QRSV_AMPL_ for a representative patient of the VV group during different VV intervals with indication of the HW value in between LV and RV pacing. The amplitude of the vector is indicated by the distance of a data point from the center, whereas the angle in a plane is indicated by its position in the *X*-*Y* and *X*-*Z* plane. *Concentric circles* indicate 1 mV (*upper row*) and 0.67 mV (*middle and lower row*s) amplitude units, respectively

During intrinsic conduction in the AV group (LBBB), the QRS vector pointed toward the back and left side of the patient with the highest amplitude in the transversal plane (Fig. 1). In contrast, during RV pacing in the VV group, the vector pointed more in the craniocaudal direction (upward). LV pacing with a short AV interval reversed the vector toward the front and right side of the patient. Changes in QRSV_AMPL_ and its orientation in the frontal and transversal plane are shown for representative patients of the AV and VV groups during all stimulation intervals (Fig. 1, lower panels).

### Effect of Different Degrees of LV and RV Pre-Excitation on Electrical, Mechanical, and Hemodynamic Parameters

**Fig. 2:**
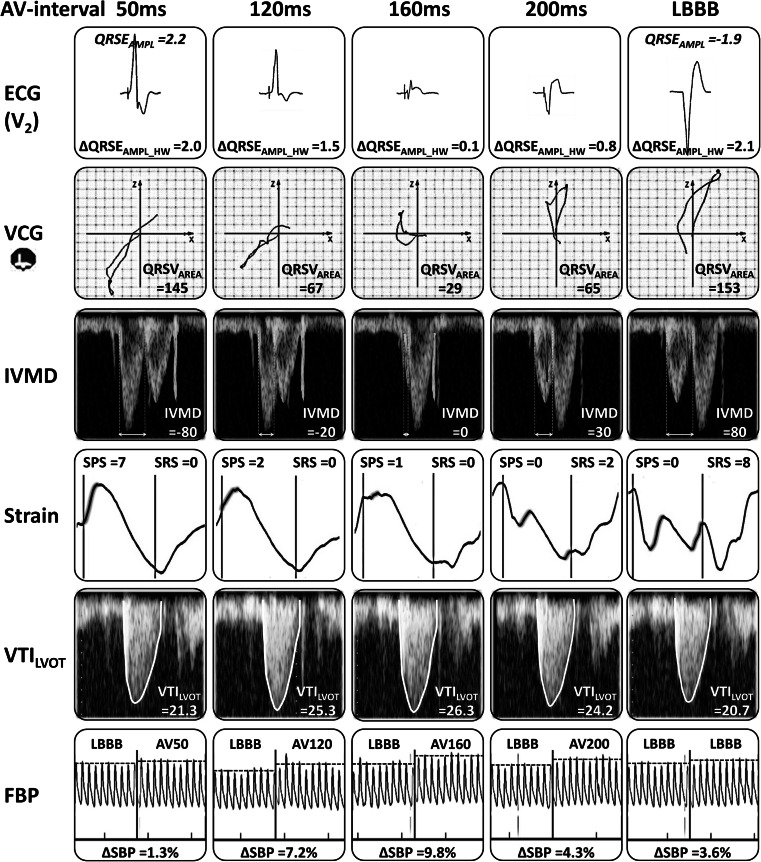
Overview of the electrical, mechanical, and hemodynamic parameters at different AV intervals. A representative example of electrical, mechanical, and hemodynamic parameters while increasing the AV interval during LV pacing. *From top to bottom*: ECG in lead V_2_ (note that the value halfway in between LV pacing and LBBB is (−1.9 + 2.2) / 2 = 0.15), VCG in the transversal plane, pulsed wave Doppler signals over the pulmonary and aortic valve with values of the interventricular mechanical delay (IVMD), septal strain curves with values of systolic pre-stretch (SPS) and systolic rebound stretch (SRS; the interval within the vertical lines represents ystole), velocity time integral over the LV outflow tract (VTI_LVOT_), finger blood pressure (FBP) measurements just before and after the transition from intrinsic conduction to a specific AV interval (for further explanation see text in “Methods”)

In Fig. 2, an overview of all electrical (ECG and VCG), mechanical (IVMD, SPS, and SRS), and hemodynamic (VTI_LVOT_ and SBP) parameters is presented as measured at different stimulation intervals for a representative patient of the AV group. When increasing the AV interval from 50 to 160 ms, the morphology of the ECG and VCG gradually changed, characterized by a decrease of QRS amplitudes and area. By further prolongation of the AV interval, these variables increased again, yet showing an opposite direction. IVMD started with earlier aortic ejection when applying LV pre-excitation, gradually becoming more synchronous and ending with earlier pulmonary ejection when loosing LV capture. With short AV intervals, there was considerable SPS, indicating passive early systolic septal stretch caused by early LV free wall activation, which gradually decreased and disappeared with longer AV intervals. SRS was absent during LV pre-excitation and increased with prolonging AV intervals. VTI_LVOT_ and SBP increased when increasing AV interval from 50 to 160 ms and subsequently decreased again.

### Prediction of the Optimal AV and VV Interval with Vectorcardiography

**Fig. 3:**
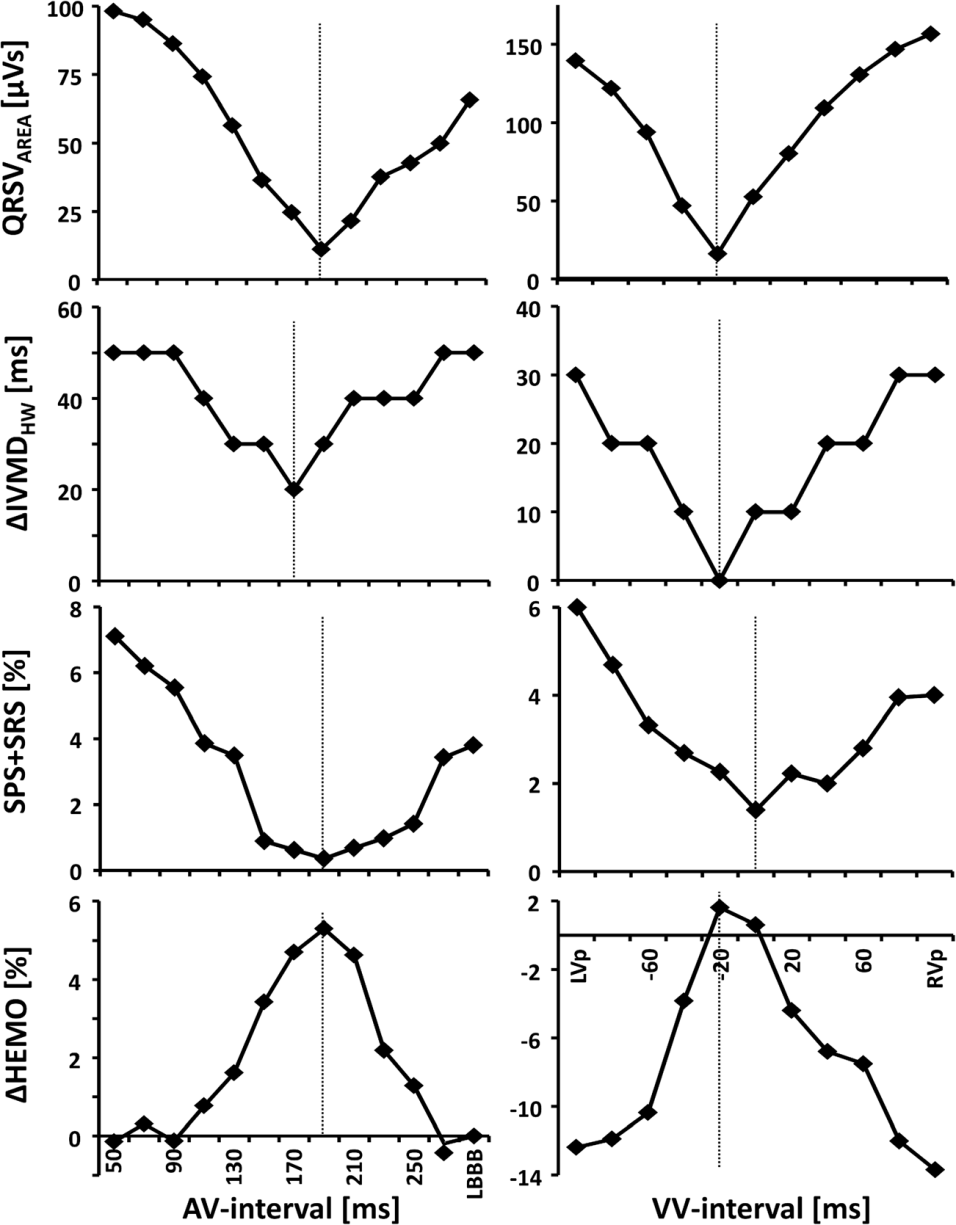
Change in electrical, mechanical, and hemodynamic parameters during different stimulation intervals. Presented are QRS vector area (QRSV_AREA_) and interventricular mechanical delay (∆IVMD_HW_) with their values halfway in between those during LV and RV pre-excitation, total septal systolic stretch (SPS + SRS), and the hemodynamic increase relative to baseline LBBB (∆HEMO) as a function of the paced AV interval (*left*) and VV interval (*righ*t) in two representative patients. Note that the stimulation interval with smallest QRSV_AREA_ corresponds closely with that of lowest ∆IVMD_HW_ and SPS + SRS, and highest ∆HEMO

**Fig. 4:**
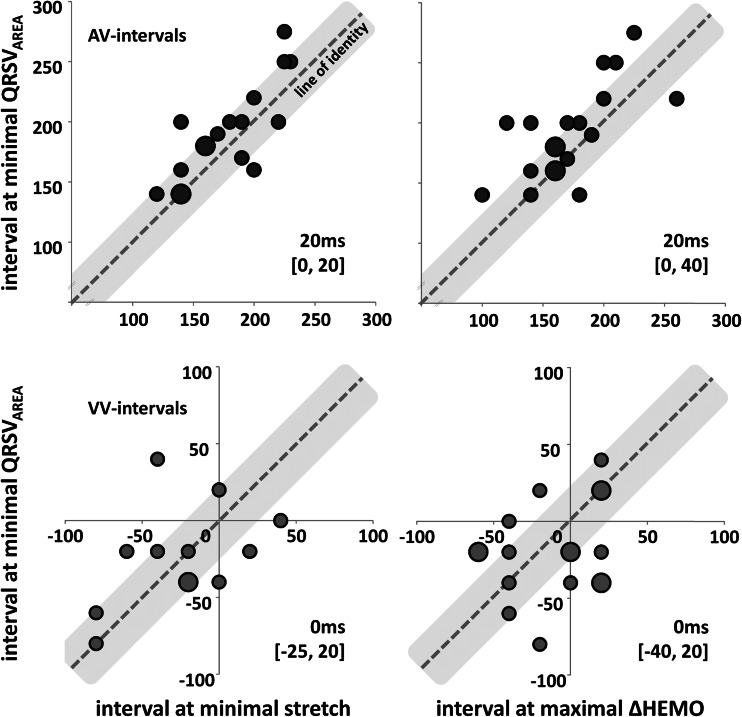
Agreement between VCG, mechanical, and hemodynamic predicted stimulation intervals. Correlation between the AV (*upper row*) and VV (*lower row*) intervals with smallest QRSV_AREA_ compared to that with lowest systolic stretch (*left*) and with highest ∆HEMO (*right*). Shown is the line of identity (*X* = *Y*) and the range within 20 ms of this line. The *area of the dots* indicates the number of observations at that specific value; clustering of data points are due to stepwise increase of AV and VV intervals

Plotting QRSV_AREA_ as a function of AV or VV interval showed a parabolic curve with a distinct minimum in the middle (Fig. 3). At the stimulation interval with smallest QRSV_AREA_, systolic stretch was lowest and hemodynamic improvement was largest, also for the entire group (Fig. 4, Table 2). Combining data from the AV group and VV group, the smallest QRS vector area identified stimulation intervals with minimal systolic stretch (median difference 20 ms [IQR −20, 20 ms]) and maximal hemodynamic response (10 ms [−20, 40 ms]).

**Table 2:** Agreement between QRSV_AREA_ and ∆QRSE_AMPL_HW_ with systolic stretch and hemodynamic function for prediction of optimal CRT settings

Prediction: Bias ± LoA	lowest QRSV_AREA_	lowest ∆QRSE_AMPL_HW_
Lowest systolic stretch		
AV-intervals (ms)	13 ± 48	−2 ± 47
VV-intervals (ms)	2 ± 72	−17 ± 70
total (ms)	8 ± 59	−8 ± 58
Highest ∆HEMO		
AV intervals (ms)	19 ± 60	7 ± 56
VV intervals (ms)	−7 ± 72	−-26 ± 65
Total (ms)	7 ± 70	−8 ± 68

Presented are bias with limits of agreement (LoA); defined as mean difference ±1.96*SD

### Prediction of the Optimal AV and VV Interval with Electrocardiography

**Fig. 5:**
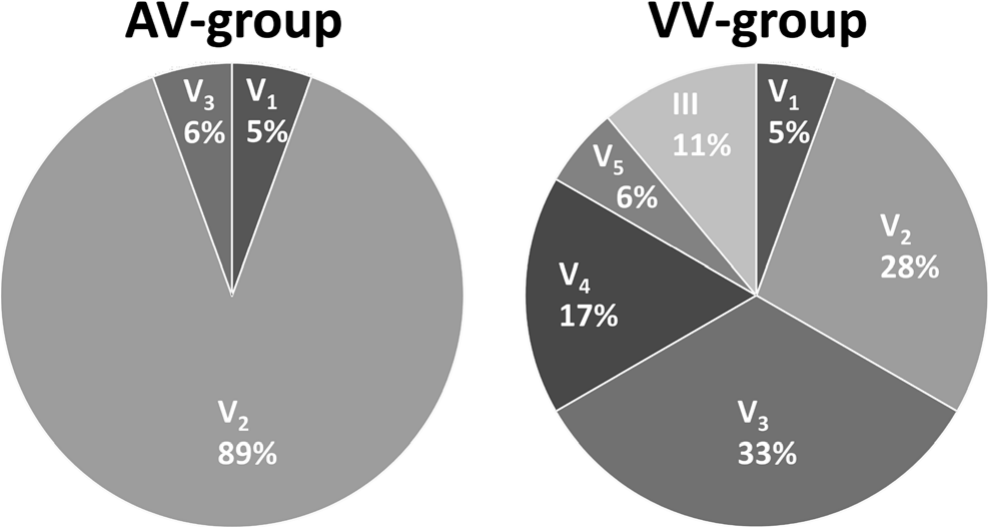
Distribution of ECG lead_MAX_. Pie plots indicating the distribution of ECG leads with largest difference in QRS amplitude between LV pacing and LBBB for patients in the AV group (*left*) and between LV and RV pacing for patients in the VV group (*right*)

We reasoned that the effect of resynchronization is best appreciated and most reliably measured in the ECG lead showing the largest changes in voltage (lead_MAX_). This lead_MAX_ was predominantly found to be lead V_2_, especially in the AV group (Fig. 5). The optimal AV and VV interval, predicted from the setting that results in a QRSE_AMPL_ closest to the value halfway between LV pacing and LBBB or RV pacing (lowest ∆QRSE_AMPL_HW_) provided a prediction of the interval with lowest systolic stretch and highest ∆HEMO that was comparable to that of the smallest QRSV_AREA_ (Table 2).

### The Hemodynamic Effect of Stimulation Interval Optimization

Compared to a nominal AV interval of 120 ms, ∆HEMO increased by 2.3 % [IQR 0.7–7.3 %] points at the best AV interval, with an increase of more than 5 % occurring in 35 % of patients. Similarly, compared to simultaneous BiV pacing, sequential pacing caused a 2.3 % [IQR 0.5–4.3 %] point increase in ∆HEMO with an increase of more than 5 % in 22 % of patients.

### Reliability of the Vectorcardiographic Measurements

The goodness of fit of raw data points with a fitted sixth order polynomial curve at various AV and VV intervals was highest for QRSV_AREA_, QRSV_AMPL_, and QRSE_AMPL_ (*R* ^2^ = 0.99; 0.98; 1.00), good for IVMD, SPS, and SRS (*R* ^2^ = 0.98; 0.97; 0.96), and lowest for ∆HEMO (*R* ^2^ = 0.87). The high *R* ^2^ value for the electrical parameters indicates minor noise in these measurements, producing a good reliability.

## Discussion

This small study in CRT patients proves the principle that electrical vectorial forces, derived from 3D VCG or the 12-lead ECG, can predict the AV and VV intervals that lead to optimal cardiac mechanical and hemodynamic function. This signifies a strong potential of these electrical measurements for routine optimization of the AV and VV intervals in CRT, especially since the method is easy to perform and has a low measuring variability.

### Vectorcardiography as an AV and VV Optimization Tool

While most methods for CRT optimization are based on cardiac mechanic or hemodynamic measurements, some studies show that also QRS duration can be used to this purpose, the narrowest QRS complex predicting the largest hemodynamic benefit [19, 24]. It is plausible that indicators of QRS morphology, like QRS area, are more sensitive and robust predictors of good resynchronization than QRS duration, because the former are based on the entire QRS complex in all leads. This is reminiscent of the better prediction of CRT response by QRS morphology (i.e., LBBB morphology) than by QRS duration alone [25].

Individual determination of maximal cancelation of electrical forces seems important in the light of observations made by noninvasive electrical mapping (ECG imaging), showing that lines of block may appear or disappear during certain pacing configuration in some patients [26, 27]. The latter observations also indicate why measured markers of electrical dyssynchrony may be preferred over the ones that are predicted based on EGMs measured during intrinsic rhythm as used by several commercial optimization algorithms [5, 12, 14].

The observation that the VCG approach worked equally well in LBBB and AV block patients indicates that intrinsic and paced LBBB activation follow similar rules for resynchronization.

Values of the optimal AV interval in the present small study (120–220 ms) appear longer than those observed in other studies [5]. However, it should be kept in mind that in the presently investigated optimization during single site LV pacing, optimal resynchronization requires fusion with the intrinsic activation wavefront.

The use of an AV (and VV) optimization tool that solely focuses on electrical optimization (“resynchronization”) seems to ignore the effect of timing on filling of the ventricles. However, both animal and human studies demonstrate that in the range of AV intervals used for CRT, the effect of the AV interval on filling is small [28, 29]. Nevertheless, patients with a restrictive filling pattern and high LV filling pressures prior to CRT may depend more on optimal filling [30].

VCG can easily be applied in clinical practice. In the present study, we used a dedicated 3D VCG system. However, most commercially available ECG machines have algorithms to construct VCGs using the inverse Dower or Kors’ regression transformation [31, 32]. This approach may provide additional information on top of reading the 12 leads (like automatic calculated QRS area), but the accuracy of the inverse transformations has yet to be demonstrated for CRT patients.

### Electrocardiography as an AV and VV Optimization Tool

Clinical application is even easier when using the 12-lead ECG, in particular the lead that shows the largest change in amplitude between LV pacing and LBBB or RV pacing (“lead_MAX_”). This can be explained by the observation that this lead (most commonly V_2_–V_3_) is the strongest determinant of the direction and amplitude of the entire 3D VCG (mostly antero-posterior and apico-basal forces in these patients with LBBB and RV pacing, respectively). While VCG may provide a more comprehensive image, “lead_MAX_” may be easiest to use in clinical practice.

Some physicians use the setting with the smallest QRS duration as a practical optimization method. Recently, this approach was shown to improve acute hemodynamic response compared to nominal AV and VV programming in CRT patients [19]. In our previous animal study, QRS duration appeared to be a worse predictor of the optimal AV and VV interval than the QRS vector amplitude halfway in between LV and RV pacing [15]. An explanation can be that although QRS duration reflects total ventricular activation time, it correlates only poorly with electrical and mechanical interventricular dyssynchrony [15, 28, 33]. Moreover, measuring the QRS duration is a precise task which requires at least an ECG paper speed of 50 mm/s and measurement with digital cursors [19]. Instead, QRS amplitude and area are relatively easy to measure and changes in these variables are larger, making them more sensitive to changes in dyssynchrony.

### Reliability of Measurements

Measurements of QRS area by VCG and QRS amplitude by ECG were robust, as indicated by the high value for goodness of sixth order polynomial fitting (*R* ^2^ = 0.99 and *R* ^2^ = 1.00, respectively). This was considerably better than for VTI_LVOT_ (*R* ^2^ = 0.88), the usual approach for VV optimization in clinical practice. This relatively low value for VTI was found even despite the use of six averaged beats in our protocol. These findings are in agreement with our previous animal experimental study where vectorcardiographic measurements were highly reproducible with a good signal to noise ratio [15]. The importance of robust and reproducible measurements is highlighted by the search for one specific AV or VV interval to select in CRT optimization. This means that the optimum of a curve over different AV or VV intervals could be at a completely different setting when repeating all AV or VV intervals in case of low reproducibility of the measurement (whether because of noise in the measurement or because of physiological influences like loading of the heart, breathing, autonomic nerve system). Avoiding this problem by using a more reproducible measurement as VCG and ECG (less noise, less physiological influence), the optimum can be chosen with confidence. Poor reproducibility of the VTI_LVOT_ measurement [9, 10] may explain why the SMART-AV study was not able to show a benefit using this approach for CRT optimization [5].

### Future Implications

The results from this study indicate that the use of VCG or ECG for AV and VV optimization is highly promising. This way of optimization makes it more readily available in all centers and feasible to perform even routinely at follow-up visits, which may be important since remodeling of the heart may change optimal settings over time. The results strongly encourage to perform a larger clinical trial to further support the benefit of ECG and VCG for CRT optimization.

### Limitations

This is a relatively small proof of principle study, which should stimulate to perform a large prospective randomized trial. The small number of patients included may bear the risk of selection of patients. However, Table 1 shows that the patients studied are fairly representative for a CRT population.

The present study only investigated the acute hemodynamic response to CRT. A good acute response does not necessarily imply good long-term outcome [34], so future studies should also address long-term outcome.

We only investigated the use of VCG and ECG for CRT optimization of patients with a right-to-left ventricular activation pattern (LBBB and RV pacing) because such pattern is most amenable to CRT. The results of this study may not apply to right bundle branch block or intraventricular conduction disturbances, but indication for CRT regarding these conduction disturbances is discouraged by current guidelines [35].

While VCG was digitally acquired, ECG measurements were performed manually, which might have been less accurate than using digital calipers. However, by performing them manually, the results of this study can be directly translated in routine clinical practice.

## Conclusion

This study proves the principle that the QRS vector obtained from 3D VCG or from the ECG lead with largest voltage changes is a practical and reliable tool for optimizing stimulation intervals in CRT patients. Larger studies are needed to show whether these measures can be used for routine practice in this field.
